# Assessment of the Accuracy of Clear Aligner Therapy for Maxillary Expansion: A Prospective Clinical Study

**DOI:** 10.2147/CCIDE.S586073

**Published:** 2026-03-25

**Authors:** Puja Kumari, Rajiv Ahluwalia, Tina Chugh, Nishant Gupta, Mayank Gupta, Waqas Sami, Moattar Raza Rizvi

**Affiliations:** 1 Department of Orthodontics and Dentofacial Orthopedics, Santosh Dental College, Santosh Deemed to be University, Ghaziabad, India; 2 Department of Pre-Clinical Affairs, College of Nursing, QU Health, Qatar University, Doha, Qatar; 3 Faculty of Allied Health Sciences, Santosh Deemed to be University, Ghaziabad, Delhi NCR, 201009, India

**Keywords:** clear aligner therapy, maxillary expansion, transverse dimension, expansion accuracy, digital orthodontics

## Abstract

**Background:**

Clear aligner therapy (CAT) is increasingly used for transverse dentoalveolar expansion of the maxillary arch; however, the extent to which digitally planned movements are clinically expressed remains uncertain.

**Objective:**

To evaluate the accuracy of maxillary transverse expansion achieved with clear aligners by comparing digitally predicted changes with clinically measured outcomes.

**Methods:**

Twelve skeletally mature adults with Class I malocclusion and maxillary constriction underwent 16 weeks of CAT. Transverse widths were recorded at pretreatment, digitally planned, and post-treatment stages using a standardized three-dimensional digital workflow with calibrated measurements on high-resolution printed models. Measurements included intercanine, inter-premolar, intermolar (mesial and distal cusp), and trans-palatal landmarks. Accuracy was calculated as the percentage of achieved relative to planned expansion. Paired-sample *t*-tests and repeated-measures ANOVA were performed (α = 0.05).

**Results:**

All transverse dimensions increased following treatment. Intercanine width increased by 1.59 mm, while first and second inter-premolar widths increased by approximately 1.21 mm. Posterior expansion was comparatively smaller, with first and second-molar widths increased by 0.7–1.0 mm. Overall accuracy ranged from 45.1% to 63.0%, indicating partial expression of digitally planned movements. Predictability was higher in the anterior and premolar regions (approximately 56–61%) than the posterior and trans-palatal sites (approximately 49–52%). Expansion remained symmetrical, with no significant right-left differences in buccolingual inclination. No clinically detectable adverse effects were observed during the study period. Males demonstrated slightly greater second-molar transverse dimensions (p<0.05).

**Conclusion:**

Clear aligner therapy produced measurable and symmetrical dentoalveolar maxillary expansion in skeletally mature adults, however the magnitude of transverse change was consistently lower than digitally planned values. Predictability decreased posteriorly, suggesting that calibrated digital overcorrection may improve alignment between virtual planning and clinical expression.

## Plain Language Summary

Clear aligners are popular orthodontic devices because they are comfortable, nearly invisible, and help patients maintain good oral hygiene. In addition to straightening teeth, orthodontists now use aligners to widen the upper dental arch in patients with a narrow upper jaw, a condition called maxillary constriction. A narrow upper jaw can lead to crowding, crossbites, breathing difficulties, and bite imbalance. Although digital software allows clinicians to plan expansion precisely, understanding how accurately aligners achieve these planned movements in real patients remains important.

In this study, we followed 12 young adults who used clear aligners for 16 weeks to widen their upper jaws. We recorded digital scans before treatment, after digital planning, and after 16 weeks of aligner use. We measured several distances between the canines, premolars, and molars to compare the expansion predicted by the software with the expansion achieved clinically.

All participants showed measurable widening of their upper teeth. Clear aligners produced measurable expansion, although the achieved widening was lower than digitally planned. On average, patients achieved 45%–63% of the intended expansion. The front teeth (canines and premolars) responded more predictably than the back teeth (molars). Most of the widening occurred through controlled outward tipping of the teeth rather than full bodily movement within the bone. No clinically detectable adverse effects on the gums or teeth were observed during the study period.

## Introduction

Transverse maxillary deficiency (TMaxD) represents one of the most frequent dentofacial deformities encountered in orthodontics, manifesting as arch constriction, unilateral or bilateral posterior crossbite, and anterior crowding that can compromise occlusal function, airway patency, and facial harmony.^1^,^2^ The prevalence of transverse discrepancies in adults is increasing due to untreated transverse constrictions and long-standing dentoalveolar compensations.^3^ Such deficiencies are not purely esthetic; they adversely affect masticatory efficiency and nasal airflow and are associated with increased periodontal strain and relapse potential following orthodontic treatment.^4^ Therefore, correction of maxillary constriction remains a cornerstone of orthodontic therapy aimed at restoring functional stability and promoting balanced craniofacial development.^5^

Historically, several mechanical approaches have been developed to address TMaxD.^6^ Conventional slow maxillary expansion (SME) applies light, continuous forces that promote dentoalveolar widening and minimize tissue trauma, whereas rapid maxillary expansion (RME)^7^ uses heavy intermittent forces to separate the mid-palatal suture, producing greater skeletal movement in growing patients.^7^,^8^ Although RME is highly effective in children and adolescents, its skeletal efficacy diminishes after puberty due to increasing sutural interdigitation.^9^ In skeletally mature adults, surgically assisted rapid palatal expansion (SARPE) or mini-screw-assisted rapid palatal expansion (MARPE) is frequently recommended to overcome sutural resistance.^10^ However, these procedures are invasive, technique-sensitive, and associated with postoperative discomfort, potential periodontal risks, and the need for specialized surgical infrastructure.^11^ The limitations of traditional appliances such as Haas or Hyrax expanders-including bulkiness, difficulty in maintaining oral hygiene, and compromised esthetics, have driven the development of more patient-friendly, digitally integrated alternatives.^12^

Clear aligner therapy (CAT) has transformed orthodontics by integrating digital treatment planning with patient-centered esthetics.^13^ Initially limited to mild crowding and spacing correction, advances in computer-aided design/manufacturing (CAD/CAM) have broadened CAT’s indications to include arch form modification, intrusion, distalization, and limited expansion.^14^ The aligner system allows incremental tooth movement through programmed staging and attachment-guided force application while maintaining oral hygiene and comfort advantages over fixed appliances.^15^ Nevertheless, the biomechanical predictability of complex movements, particularly bodily translation, torque control, and anchorage maintenance remains less reliable than with conventional brackets and wires.^16^ This limitation is especially pertinent in transverse expansion, where aligners primarily produce dentoalveolar widening through controlled buccal tipping rather than skeletal remodeling.^17^ In skeletally mature adults, true skeletal expansion requires midpalatal suture separation, which cannot be achieved with conventional aligner mechanics. Accordingly, transverse changes observed with aligners should be regarded as dentoalveolar rather than skeletal expansion.

Multiple in vitro, finite-element, and clinical studies have evaluated the extent to which CAT can produce transverse changes.^18^ Measurable increases in intercanine and inter-premolar widths have been demonstrated following aligner treatment.^19^ Significant expansion in the canine and premolar regions, with comparatively limited molar movement, has also been confirmed.^20^ Clinically achieved expansion has been reported to under-express digitally planned movements, with discrepancies frequently observed in the range of approximately 30–50%, particularly in the posterior regions.^21^ A meta-analysis further consolidates these findings, concluding that aligners achieve approximately 60% of programmed expansion, primarily through dentoalveolar tipping rather than bodily displacement.^22^ Finite-element analyses corroborate this mechanism, demonstrating stress concentration in the alveolar cortical plate and torque under-expression in molars, thereby limiting skeletal contribution to transverse expansion.^23^

While these studies substantiate CAT’s ability to expand arches within mild to moderate limits, the predictability and regional variability of movement remain subjects of ongoing investigation.^24^ Expansion appears most efficient in the premolar region (approximately ∼70%) but least effective in second molars (often below 50%), suggesting a gradient of response from anterior to posterior teeth.^25^ Significant discrepancies between virtual setups and clinically achieved outcomes further emphasize the potential need for digital overcorrection to compensate for aligner elasticity and limitations in torque control.^19^ Despite these insights, most investigations have been retrospective, device-specific, or limited to adolescent populations, restricting extrapolation to skeletally mature adults, where sutural ossification and reduced bone plasticity may further limit dentoalveolar expansion efficacy.^26^,^27^

Despite the rapid adoption of digital orthodontic workflows, the translation from digitally planned expansion to clinically realized outcomes in adults remains insufficiently quantified.^28^ Contemporary orthodontic practice increasingly relies on fully digital treatment pathways, including intraoral scanning, three-dimensional virtual setup simulations, and CAD/CAM-based appliance fabrication, particularly in clear aligner therapy. As virtual treatment planning now directly governs aligner staging and tooth movement prescription, validating the clinical expression of digitally programmed transverse movements has become essential for evidence-based orthodontic care.^29^,^30^

Few prospective human studies have systematically compared digitally planned and clinically achieved transverse changes using standardized three-dimensional (3D) measurement protocols.^31^ Moreover, the extent to which expansion accuracy varies by tooth region, patient sex, or side symmetry remains inadequately characterized. As digital treatment planning increasingly governs aligner staging and overcorrection, quantifying this discrepancy is essential for clinical predictability and for guiding evidence-based aligner design.^24^,^32^ Understanding where and why under-expression occurs can refine digital protocols, reduce mid-course refinements, and improve overall treatment efficiency-key considerations in both clinical and academic allied health settings.

Therefore, this prospective clinical study aimed to evaluate the accuracy and clinical expression of clear aligner therapy in producing maxillary expansion in adults by comparing digitally planned transverse changes with clinically achieved outcomes after 16 weeks of treatment. The working hypothesis was that the expansion achieved with clear aligners would be significantly lower than digitally planned values across all measured sites, with accuracy decreasing progressively from anterior to posterior regions due to greater biomechanical resistance. Unlike previous retrospective or mixed-age investigations, the present study prospectively evaluates transverse expansion accuracy exclusively in skeletally mature adults using a standardized three-dimensional digital workflow with calibrated model validation and without adjunctive expansion mechanics.

## Materials and Methods

### Study Design

This prospective longitudinal clinical study was conducted in the Department of Orthodontics and Dentofacial Orthopedics, Santosh Deemed to be University, Ghaziabad, India, between January 2023 and May 2024. The study followed a within-subject design comparing digitally planned maxillary transverse expansion with clinically achieved outcomes after 16 weeks of clear aligner therapy. The study was designed and reported in accordance with the STROBE (Strengthening the Reporting of Observational Studies in Epidemiology) guidelines.

### Sample Size Calculation

An a priori power analysis was performed using G*Power version 3.1.9.4 (Heinrich Heine University, Düsseldorf, Germany) to determine the minimum required sample size for a paired-sample *t*-test (two-tailed). In the absence of pilot data, a large effect size (Cohen’s d = 0.8) was conservatively assumed based on previously published evidence demonstrating clinically meaningful discrepancies between digitally planned and clinically achieved transverse expansion with clear aligners.^24^ Assuming α = 0.05, and a statistical power of 0.82, the minimum sample was 12 participants (δ = 2.77; critical *t* = 1.80). To account for potential attrition, 13 participants were initially recruited, and 12 completed the 16-week study.

### Participant Selection

Participants were recruited consecutively from adults presenting with Angle Class I malocclusion, mild-to-moderate maxillary crowding (2–6 mm), and transverse maxillary constriction with or without posterior crossbite. Crowding severity was quantified as tooth-size arch-length discrepancy (TSALD), calculated by subtracting the sum of the mesiodistal tooth widths (canine to first molar) from the available maxillary arch perimeter measured on standardized digital models using calibrated linear measurement tools.^33^ Eligible participants were required to have a healthy periodontium, satisfactory oral hygiene, and documented compliance with 20–22 hours of daily aligner wear.

Exclusion criteria included systemic diseases or medications affecting bone turnover, craniofacial anomalies, skeletal discrepancies requiring surgical correction, active periodontal disease, previous orthodontic treatment, temporomandibular disorders, parafunctional habits (including bruxism or clenching), and severe occlusal wear that could compromise landmark identification.

Only skeletally mature adults were enrolled to ensure that observed transverse changes primarily reflected dentoalveolar adaptation rather than skeletal expansion. Maxillary constriction was diagnosed clinically based on transverse discrepancy between the maxillary and mandibular arches, presence or absence of posterior crossbite (unilateral or bilateral), and arch form evaluation on digital models.^34^ Because cone-beam computed tomography was not performed, differentiation between purely skeletal and dentoalveolar components was based on clinical examination and dental cast analysis. Baseline buccolingual inclination of posterior teeth was recorded to assess initial torque conditions prior to expansion.

### Digital Planning and Treatment Protocol

Clear aligners were fabricated in-house using a CAD/CAM workflow. Digital treatment planning was performed with 3Shape Clear Aligner Studio (3Shape A/S, Copenhagen, Denmark), and stereolithographic models were printed to enable thermoforming of aligners. Transverse expansion was programmed exclusively through aligner-driven mechanics without the use of auxiliary appliances, cross-arch elastics, or skeletal anchorage devices. Composite attachments were placed where required to enhance retention and force expression.

**Figure 1:**
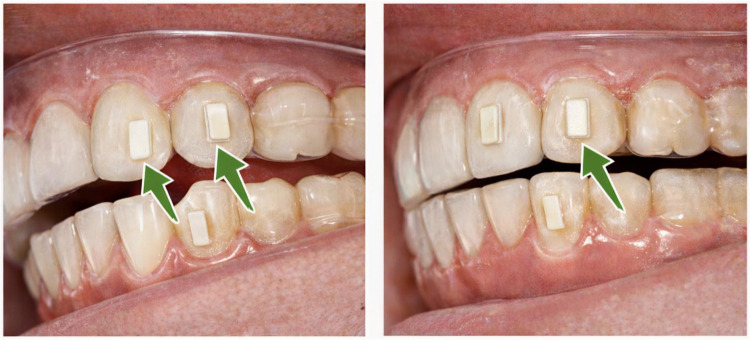
Representative illustration of in-house composite attachment design and placement for transverse expansion. Rectangular composite attachments in vertical and horizontal orientations were selectively bonded to premolars and molars to enhance aligner retention and optimize force expression during buccal expansion.

Attachment design was customized according to individual tooth morphology and planned movement requirements. Rectangular composite attachments in both vertical and horizontal orientations were selectively placed on premolars and molars to enhance aligner retention and optimize force expression during buccal expansion. Because the aligners were fabricated in-house, attachment geometry, size, and positioning were manually designed within the digital setup rather than generated using proprietary commercial presets (Figure 1).

The virtual setup (T_2_p) prescribed approximately 0.25 mm of buccal displacement per side per stage, individualized according to each patient’s arch form and crowding severity. Expansion was programmed as controlled buccal movement without intentional root torque overcorrection or additional bodily translational mechanics. No programmed overcorrection was incorporated in the digital setup to allow unbiased evaluation of intrinsic expansion accuracy.

Aligners were thermoformed from 0.75 mm medical-grade thermoplastic sheets under positive pressure over stereolithographically printed models. Each aligner was worn for 14 days for 20–22 hours per day. The 16-week evaluation period corresponded to completion of eight aligner stages under the 14-day change protocol, providing a standardized interval for assessing early clinical expression of programmed transverse movements.^35^ Patients were evaluated biweekly to assess aligner fit, attachment integrity, and compliance. No adjunctive forces were used to maintain methodological uniformity.

### Measurement Time Points

Three standardized stages were analyzed to quantify transverse expansion. The baseline (T1) scan represented pretreatment conditions and was captured using a *Trios 3* intraoral scanner (3Shape A/S, Copenhagen, Denmark). The planned (T2p) model, generated in 3Shape Clear Aligner Studio, represented the digitally predicted outcome after 16 weeks of treatment. The achieved (T2a) model documented the post-treatment state following 16 weeks of aligner wear and was obtained using identical scanning parameters.

**Figure 2:**
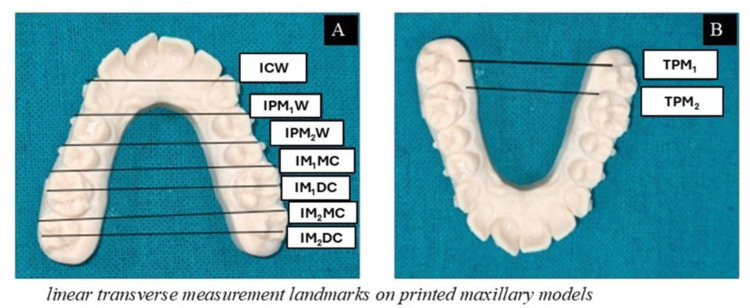
Standardized transverse measurement landmarks on maxillary models. (**A**) Linear dental arch width measurements including intercanine width (ICW), first and second inter-premolar widths (IPM_1_W, IPM_2_W), and mesial and distal cusp widths of the first and second molars (IM_1_MC, IM_1_DC, IM_2_MC, IM_2_DC). (**B**) Trans-palatal widths at the first and second molars (TPM_1_, TPM_2_) were used to assess changes within the palatal vault. Reference points and measurement planes are illustrated for standardization.

**Figure 3:**
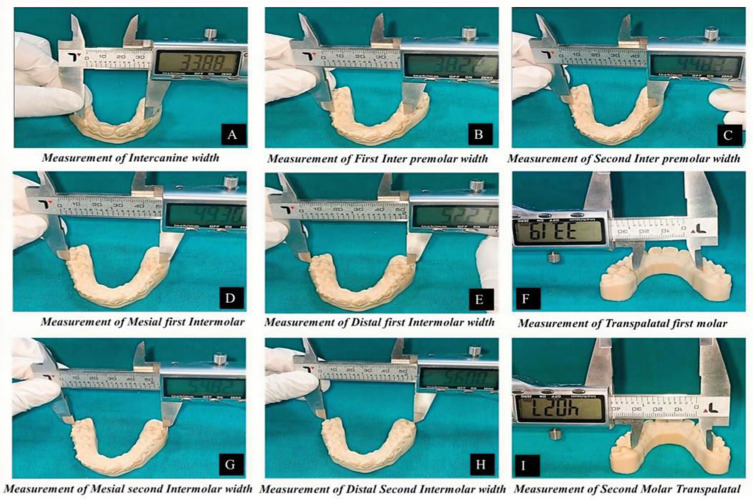
Standardized linear transverse width measurement protocol on printed maxillary models using a calibrated digital vernier calliper (accuracy ±0.01 mm). (**A**) Intercanine width (ICW). (**B**) First inter-premolar width (IPM_1_W). (**C**) Second inter-premolar width (IPM_2_W). (**D**) First molar mesial cusp width (IM_1_MC). (**E**) First molar distal cusp width (IM_1_DC). (**F**) First molar trans-palatal width (TPM_1_). (**G**) Second molar mesial cusp width (IM_2_MC). (**H**) Second molar distal cusp width (IM_2_DC). (**I**) Second molar trans-palatal width (TPM_2_). Measurements were recorded at predefined anatomical landmarks.

All digital models were exported in STL format and printed on a Form 3B stereolithographic 3D printer (Formlabs, USA; 50 µm layer resolution) as part of the standardized three-dimensional workflow with calibrated model validation to verify dimensional fidelity, with deviations maintained within ±0.05 mm. The reliability and dimensional accuracy of measurements obtained from scanned and printed models have been previously validated, demonstrating no clinically significant differences compared with conventional plaster models.^36^ Uniform scanning conditions and a single operator minimized acquisition bias. The anatomical landmarks used for standardized transverse measurements are illustrated in Figure 2, and the linear measurement protocol on printed models is shown in Figure 3.

### Measurement Parameters

**Figure 4:**
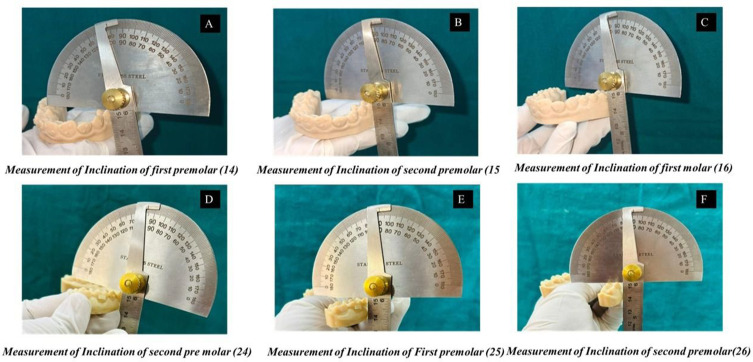
Representative illustration of buccolingual inclination measurement on printed maxillary models. (**A–C**) Maxillary right posterior teeth (FDI: 14, 15, 16). (**D–F**) Maxillary left posterior teeth (FDI: 24, 25, 26). The long axis of each tooth was determined using standardized anatomical landmarks, and the inclination angle (°) was measured as the angle formed between the tooth long axis and a perpendicular reference to the occlusal plane. Measurements were recorded to quantify dentoalveolar tipping associated with transverse expansion.

Linear transverse measurements were recorded using a digital vernier caliper (Mitutoyo 500–196-30, Japan; measurement accuracy ±0.01 mm). The following transverse widths were assessed: intercanine (ICW), first and second inter-premolar (IPM1W, IPM2W), and the mesial and distal cusp widths of the first and second molars (IM1MC, IM1DC, IM2MC, IM2DC). Anatomical reference landmarks for all measurements are illustrated in Figure 2. Trans-palatal widths at the first and second molars (TPM1, TPM2) were additionally recorded to evaluate changes in palatal vault dimension. Buccolingual inclination of the first and second premolars and first molars was evaluated bilaterally on printed models. Inclination was defined as the angle formed between the long axis of each tooth and a perpendicular reference to the occlusal plane, determined using standardized anatomical landmarks. The buccolingual inclination measurement protocol is illustrated in Figure 4

### Measurement Reliability

All measurements were recorded by a single calibrated examiner to minimize inter-observer variability. Intra-examiner reliability was assessed by re-measuring five randomly selected models after a two-week interval. Reproducibility was evaluated using the Intraclass Correlation Coefficient (ICC; two-way mixed-effects model, absolute agreement). ICC values greater than 0.90 were considered indicative of excellent reliability.^37^ The examiner was blinded to participant identity and measurement time points throughout the assessment process.

### Calculation of Expansion and Accuracy

Planned expansion was defined as the difference between the digitally predicted model (T_2_p) and baseline (T_1_), whereas achieved expansion represented the difference between the post-treatment scan (T_2_a) and baseline (T_1_). Clinical accuracy for each transverse dimension was calculated using the standardized formula: Accuracy (%) = (T_2_a-T_1_)/(T_2_p-T_1_) × 100. Negative values were interpreted as contraction. Accuracy measures for all transverse parameters (ICW, IPM1W, IPM2W, IM1MC, IM1DC, IM2MC, IM2DC, TPM1, TPM2) reflected the extent to which digitally prescribed movements were reproduced clinically. Comparative visualization of anatomical landmarks and transverse measurements is presented in Figures 2 and 3, while buccolingual inclination assessment is illustrated in Figure 4.

### Bias Control and Data Integrity

All scans, digital setups, and measurements were performed by a single trained operator who remained blinded during data entry and analysis to minimize measurement and allocation bias. Participants demonstrating non-compliance exceeding three consecutive days of missed aligner wear were excluded from analysis. No auxiliary expansion devices, cross-arch elastics, or adjunctive mechanics were used to avoid confounding effects. Standardized oral-hygiene instructions were provided to all participants, and no adverse events or periodontal complications were recorded. All STL files, datasets, and measurement sheets were archived in the institutional repository (Ref. SU-ORTHO-DATA-CAT-2024) to ensure traceability and facilitate external verification.

### Outcome Measures

The outcome framework was designed to evaluate both quantitative accuracy and the biomechanical pattern of aligner-induced transverse expansion. The primary outcome was the *accuracy of maxillary transverse expansion*, defined as the percentage ratio between clinically achieved and digitally planned expansion at each transverse landmark. Linear inter-canine, inter-premolar, and inter-molar distances were analyzed to assess the fidelity of digital treatment planning. Mean differences (T_2_p - T_2_a), 95% confidence intervals, and Cohen’s *d* effect sizes were calculated to determine the magnitude and clinical relevance of discrepancies.

Secondary outcomes included the change in buccolingual inclination (°) of posterior teeth, reflecting the balance between bodily translation and tipping; Pearson correlation coefficients between planned and achieved expansions, indicating treatment predictability; anterior-to-posterior gradient analysis to evaluate regional efficiency; symmetry and sex-based variations; and functional expansion threshold analysis, defined as a ≥ 1.5 mm mean increase in intermolar width, selected based on previously reported^38^ clinical principles indicating that approximately 1.5 mm of additional intermolar expansion is typically required to establish normal posterior buccal overjet and achieve clinically meaningful transverse correction. This multifactorial design provided a comprehensive evaluation of aligner precision, predictability, and biomechanical translation.

### Statistical Analysis

Data were compiled in Microsoft Excel 2021 and analyzed using IBM SPSS Statistics (Version 26.0; IBM Corp., Armonk, NY, USA). Normality of continuous variables was assessed using the Shapiro–Wilk test, and homogeneity of variances was evaluated using Levene’s test. Descriptive statistics were reported as mean ± standard deviation (SD) with 95% confidence intervals. Planned (T_2_p) and achieved (T_2_a) transverse widths were compared using paired-sample *t*-tests. Repeated-measures ANOVA with Bonferroni adjusted post-hoc comparisons was applied to evaluate differences across T_1_, T_2_p, and T_2_a, controlling for potential inflation of type I error resulting from multiple comparisons. Pearson correlation coefficients were calculated to assess associations between planned and achieved expansions. Cohen’s *d* was used to quantify effect sizes (small = 0.2, medium = 0.5, large ≥ 0.8). Sex-based and side-based differences were analyzed using independent-samples *t*-tests. Statistical significance was set at p<0.05.

## Results

### Baseline Demographic Characteristics

A total of 12 participants completed the study. The mean age was 24.1 ± 3.9 years (range, 20–30 years). The sample included 5 males (41.7%) and 7 females (58.3%). Detailed demographic characteristics are presented in Table 1.

**Table 1:** Baseline Demographic and Clinical Characteristics (n = 12)

Characteristic	Mean ± SD	Range/n (%)
Age (years)	24.1 ± 3.9	20-30
Sex		
Male	-	5 (41.7%)
Female	-	7 (58.3%)
Crowding (TSALD) (mm)^a^	4.8 ± 1.2	2.4–5.9
Crossbite Type		
Unilateral	-	9 (75.0%)
Bilateral	-	3 (25.0%)
Premolar Inclination (°)^b^	72.4 ± 4.2	66.1–79.8

**Note**: ^a^TSALD: Tooth Size Arch Length Discrepancy, measured at baseline (T1). Values restricted to 2–6 mm according to inclusion criteria. ^b^Premolar Inclination: Calculated as the mean buccolingual inclination of the first and second maxillary premolars bilaterally at T1.

Normality of the transverse measurements was assessed using the Shapiro–Wilk test. All variables demonstrated normal distribution (W range: 0.907–0.979, p > 0.05). Homogeneity of variances across timepoints was evaluated using Levene’s test, which showed no significant violations (F range: 0.003–0.802, p > 0.05). Therefore, the assumptions for parametric repeated-measures analysis were satisfied.

### Descriptive Comparison of Transverse Widths

Descriptive statistics demonstrated a consistent increase in all maxillary transverse dimensions from pretreatment (T1) to both the digitally planned (T2p) and clinically achieved (T2a) stages (Table 2). Intercanine, inter-premolar, intermolar, and trans-palatal widths exhibited positive changes. The greatest widening occurred in the canine region, where intercanine width increased by approximately 1.6 mm. Posterior and trans-palatal measurements demonstrated comparatively smaller increases. Repeated-measures ANOVA confirmed significant overall differences across T1, T2p, and T2a (p < 0.05). Clinically achieved values remained lower than digitally planned predictions.

**Table 2:** Linear Transverse Measurements at T1, T2p, and T2a

Variable	Stage	Mean ± SD	SE	95% CI Lower	95% CI Upper	*p*
ICMW	T1	32.54 ± 3.20	0.92	30.51	34.58	0.14
	T2p	35.13 ± 3.06	0.88	33.18	37.07	
	T2a	34.13 ± 3.15	0.91	32.12	36.13	
IPM1W	T1	40.67 ± 3.06	0.88	38.73	42.61	0.25
	T2p	42.83 ± 3.21	0.93	40.80	44.87	
	T2a	41.88 ± 3.17	0.92	39.86	43.89	
IPM2W	T1	45.00 ± 2.56	0.74	43.37	46.63	0.12
	T2p	47.13 ± 2.43	0.70	45.58	48.67	
	T2a	46.21 ± 2.36	0.68	44.71	47.71	
IM1MC	T1	48.25 ± 2.15	0.62	46.89	49.61	0.06
	T2p	50.38 ± 1.99	0.57	49.11	51.64	
	T2a	49.21 ± 2.12	0.61	47.86	50.55	
IM1DC	T1	50.50 ± 2.33	0.67	49.02	51.98	0.11
	T2p	52.42 ± 2.22	0.64	51.00	53.83	
	T2a	51.50 ± 1.92	0.55	50.28	52.72	
TPM1	T1	33.92 ± 2.15	0.62	32.55	35.28	0.12
	T2p	35.79 ± 2.25	0.65	34.36	37.22	
	T2a	34.83 ± 2.06	0.59	33.52	36.14	
IM2MC	T1	54.42 ± 3.23	0.93	52.36	56.47	0.35
	T2p	56.33 ± 3.23	0.93	54.28	58.38	
	T2a	55.63 ± 3.23	0.93	53.57	57.68	
IM2DC	T1	56.13 ± 2.63	0.76	54.45	57.80	0.37
	T2p	57.71 ± 2.77	0.80	55.95	59.47	
	T2a	56.96 ± 2.73	0.79	55.22	58.70	
TPM2	T1	38.67 ± 2.39	0.69	37.15	40.18	0.21
	T2p	40.50 ± 2.58	0.75	38.86	42.14	
	T2a	39.58 ± 2.43	0.70	38.04	41.13	

**Note**: *p*<0.05 is significant. T1, pretreatment; T2p, digitally planned; T2a, clinically achieved.

**Abbreviations**: ICMW, inter-canine width; IPM1W, first inter-premolar width; IPM2W, second inter-premolar width; IM1MC, first-molar mesial-cusp width; IM1DC, first-molar distal-cusp width; TPM1, first-molar trans-palatal width; IM2MC, second-molar mesial-cusp width; IM2DC, second-molar distal-cusp width; TPM2, second-molar trans-palatal width; SD, standard deviation; SE, standard error; CI, confidence interval.

### Comparative Accuracy of Planned and Achieved Expansion

Paired-sample comparisons revealed statistically significant differences between digitally planned (T2p) and clinically achieved (T2a) transverse widths at all evaluated landmarks (p < 0.05) (Table 3). Mean discrepancies ranged from 0.71 mm (IM2MC) to 1.17 mm (IM1MC). Accuracy values ranged from 45.09% to 63.04%. Anterior and premolar regions demonstrated relatively higher accuracy, whereas posterior sites exhibited comparatively greater deviation from planned expansion.

**Table 3:** Comparison of Digitally Planned (T2p) vs Clinically Achieved (T2a) Transverse Widths Using Paired-Sample *t*-Test

Variable	T2p Mean ± SD	T2a Mean ± SD	Mean Diff	95% CI Lower	95% CI Upper	*p*	Accuracy (%)
ICMW	35.13±3.06	34.13 ± 3.15	1.00	0.86	1.14	<0.001*	61.29
IPM1W	42.83±3.21	41.88 ± 3.17	0.96	0.79	1.12	<0.001*	55.76
IPM2W	47.13±2.43	46.21 ± 2.36	0.92	0.69	1.14	<0.001*	56.86
IM1MC	50.38±1.99	49.21 ± 2.12	1.17	0.88	1.45	<0.001*	45.09
IM1DC	52.42 ± 2.22	51.50 ± 1.92	0.92	0.51	1.32	<0.001*	52.17
TPM1	35.79 ± 2.25	34.83 ± 2.06	0.96	0.54	1.37	<0.001*	48.88
IM2MC	56.33 ± 3.23	55.63 ± 3.23	0.71	0.27	1.15	0.004*	63.04
IM2DC	57.71 ± 2.77	56.96 ± 2.73	0.75	0.18	1.32	0.014*	52.63
TPM2	40.50 ± 2.58	39.58 ± 2.43	0.92	0.65	1.18	<0.001*	50.00

**Note**: Mean Difference calculated as (T2p − T2a). Positive values indicate under-expression (Planned > Achieved). Accuracy (%) = (T2a − T1)/(T2p − T1) × 100. T1 = pretreatment; T2p = digitally planned; T2a = clinically achieved. *p < 0.05 indicates statistical significance.

**Abbreviations**: ICMW, inter-canine width; IPM1W, first inter-premolar width; IPM2W, second inter-premolar width; IM1MC, first-molar mesial-cusp width; IM1DC, first-molar distal-cusp width; TPM1, first-molar trans-palatal width; IM2MC, second-molar mesial-cusp width; IM2DC, second-molar distal-cusp width; TPM2, second-molar trans-palatal width; SD, standard deviation; CI, confidence interval.

### Tooth-Specific Evaluation of Expansion Accuracy

Tooth-level analysis demonstrated site-specific variability in the expression of planned expansion (Table 4). Significant under-expression was observed at teeth 14, 15, 25 and 26, with mean discrepancies (T2p − T2a) ranging from 0.75 mm to 4.63 mm (p<0.05). Teeth 16 and 24 did not show statistically significant differences between planned and achieved expansion. Premolars exhibited a more consistent pattern of under-expansion, whereas molars demonstrated greater dispersion of values, as reflected by wider standard deviations.

**Table 4:** Tooth-Specific Discrepancies Between Planned (T2p) and Achieved (T2a) Expansion

Tooth No.	Mean Diff ± SD	SE	95% CI Lower	95% CI Upper	*p* value
14	0.75 ± 0.75	0.22	0.27	1.23	0.005*
15	0.75 ± 0.81	0.23	0.24	1.26	0.008*
16	−0.71 ± 4.31	1.25	−3.46	2.04	0.58
24	1.04 ± 2.51	0.72	−0.54	2.62	0.18
25	1.58 ± 1.88	0.54	0.39	2.77	0.014*
26	4.63 ± 2.05	0.59	3.33	5.93	<0.001*

**Note**: Mean Difference calculated as (T2p − T2a). Positive values indicate under-expression (Planned > Achieved); negative values indicate over-expression. *p < 0.05 indicates statistical significance.

**Abbreviations**: SE, standard error; CI, confidence interval.

### Sex-Wise Comparison of Achieved Maxillary Transverse Dimensions

Sex-based comparison of achieved transverse widths (T2a) showed that males exhibited larger mean dimensions than females across most landmarks (Table 5). Differences in anterior and premolar regions were not statistically significant. However, posterior measurements demonstrated significant sex-related variation with males exhibiting greater second-molar mesial and distal cusp widths (p = 0.022 and p = 0.043, respectively). Trans-palatal widths showed no statistically significant sex-based differences.

**Table 5:** Sex-Based Comparison of Achieved Transverse Widths (Independent-Samples *t* Test)

Variable	Sex	n	Mean ± SD	SE	*p* value
ICMW	Male	5	35.40 ± 3.44	1.54	0.17
	Female	7	32.64 ± 2.56	0.97	
IPM1W	Male	5	43.10 ± 4.01	1.79	0.22
	Female	7	39.93 ± 4.17	1.58	
IPM2W	Male	5	47.10 ± 2.22	0.99	0.10
	Female	7	44.36 ± 3.08	1.16	
IM1MC	Male	5	50.50 ± 2.12	0.95	0.11
	Female	7	48.43 ± 1.79	0.68	
IM1DC	Male	5	53.00 ± 2.76	1.23	0.13
	Female	7	50.50 ± 2.00	0.76	
TPM1	Male	5	34.30 ± 2.17	0.97	0.52
	Female	7	35.14 ± 2.12	0.80	
IM2MC	Male	5	57.90 ± 2.77	1.24	0.022 *
	Female	7	53.57 ± 2.28	0.86	
IM2DC	Male	5	58.60 ± 2.82	1.26	0.043 *
	Female	7	55.00 ± 1.71	0.65	
TPM2	Male	5	39.90 ± 3.09	1.38	0.65
	Female	7	39.14 ± 2.08	0.78	

**Note**: T1 = pretreatment; T2p = digitally planned; T2a = clinically achieved. *p<0.05 significant.

**Abbreviations**: ICMW, inter-canine width; IPM1W, first inter-premolar width; IPM2W, second inter-premolar width; IM1MC, first-molar mesial-cusp width; IM1DC, first-molar distal-cusp width; TPM1, first-molar trans-palatal width; IM2MC, second-molar mesial-cusp width; IM2DC, second-molar distal-cusp width; TPM2, second-molar trans-palatal width. SD, standard deviation; SE, standard error; CI, confidence interval.

### Comparison of Buccolingual Inclination Between Right and Left Sides

Assessment of buccolingual inclination revealed no statistically significant right-left differences for the premolars or first molars at T2a (Table 6). Mean inclination values were closely matched between sides for all evaluated teeth. No statistically significant unilateral differences in buccolingual inclination were detected.

**Table 6:** Comparison of Buccolingual Inclination Between Right and Left Sides (Paired-Sample *t*-Test)

Variable	Side	n	Mean ± SD (°)	SE	*p* value
1ˢ^t^ Premolar	Right	12	83.92 ± 3.15	0.91	0.12
	Left	12	81.58 ± 3.87	1.12	
2ⁿ^d^ Premolar	Right	12	80.96 ± 2.37	0.68	0.12
	Left	12	78.92 ± 3.58	1.03	
1ˢ^t^ Molar	Right	12	75.71 ± 3.06	0.88	0.33
	Left	12	77.46 ± 5.17	1.49	

**Note**: *p < 0.05 indicates statistical significance.

**Abbreviations**: SD, standard deviation; SE, standard error; n, sample number.

## Discussion

The current investigation demonstrated that clear aligner therapy produced measurable maxillary transverse expansion across all evaluated landmarks, confirming its capacity to achieve controlled dentoalveolar widening. Achieved widths represented approximately 45–63% of the digitally planned setup, indicating moderate concordance between planned and clinical outcomes. Expansion was most pronounced at the canine and premolar levels, whereas posterior and transpalatal changes were less extensive. No statistically significant side-to-side discrepancies were detected, and males exhibited slightly greater posterior widths. Collectively, these findings indicate that clear aligners can produce symmetrical and measurable dentoalveolar transverse expansion; however, the magnitude of change remains partially under-expressed relative to the virtual prescription.^39^

While these findings provide insight into early transverse expression with aligners, they should be interpreted within the methodological scope of the present design. Analysis of intercanine and inter-premolar widths revealed the largest dimensional changes, suggesting that anterior segments respond more readily to aligner forces. This observation is consistent with earlier reports describing predictable anterior widening during aligner therapy.^40^ The magnitude of change, generally ranging between approximately 1.0 and 1.6 mm in this cohort, aligns with outcomes reported in comparable adult populations.^41^ Greater responsiveness in the anterior region may be influenced, in part, by reduced cortical resistance, thinner alveolar plates, and improved aligner adaptation to tooth morphology, although these factors were not directly assessed in the present study.

Tooth-level analysis further demonstrated significant under-expression at the second molar region, reinforcing reduced posterior predictability.^26^ Reduced aligner-to-tooth contact, thicker cortical bone, and less favorable moment-to-force ratios could potentially limit bodily movement and favor crown tipping. Finite-element analyses similarly demonstrate that torque loss and localized resistance in the molar region restrict lateral translation.^42^ These biomechanical considerations may partly account for the expansion gradient observed in this study, where anterior movement was expressed more fully than posterior displacement.

The overall accuracy range of 45–63% aligns with previously reported values of 35–73% for aligner-mediated transverse movement.^19^ The discrepancy between planned and achieved expansion may reflect partial elastic relaxation of thermoplastic materials and progressive force decay during aligner wear.^43^ Posterior accuracy values, particularly at the first molar region, were lower than anterior sites, emphasizing potential mechanical limitations related to reduced stiffness and load transfer.^44^ Controlled digital overcorrection of approximately 30–40% within digital setups has been suggested to compensate for predictable under-expression.^22^ The present data support this concept, indicating that calibrated overplanning may improve concordance between digital intent and clinical outcome.

The requirement for overcorrection may be partly attributable to the viscoelastic properties of thermoplastic materials, which undergo stress relaxation and progressive force decay during the wear interval, potentially leading to under-expression of programmed movements.^45^ Additionally, transverse expansion requires effective torque control to achieve bodily movement; however, limited torque expression in posterior teeth may result in buccal crown tipping rather than true translational expansion.^42^ These biomechanical factors may contribute to the discrepancy between digitally planned and clinically achieved outcomes.

No statistically significant right-to-left differences in buccolingual inclination were observed, indicating symmetrical expansion across the arch. This bilateral uniformity may reflect balanced force distribution achieved through consistent aligner thickness and staging protocols.^14^,^46^ The inclination pattern was suggestive of controlled buccal tipping with no statistically significant evidence of unilateral torque imbalance, consistent with biomechanical models attributing expansion primarily to dentoalveolar movement rather than root divergence.^42^ The absence of asymmetry may support the capacity of aligners to maintain occlusal harmony during transverse correction.

Male participants demonstrated slightly greater posterior transverse dimensions, a difference that may be associated with larger basal bone width and alveolar volume commonly reported in males.^47^ Although anterior segments exhibited comparable behavior across sexes, posterior differences suggest that underlying morphologic characteristics may influence mechanical responsiveness. Similar sex-based variations have been reported in other adult aligner studies.^19^,^48^ These findings underscore the importance of individualized digital planning that accounts for anatomic variability.

The findings of this study are consistent with existing evidence indicating that, in skeletally mature adults, clear aligners do not induce midpalatal suture separation; therefore, the transverse changes observed should be interpreted as dentoalveolar expansion rather than skeletal widening. Given the material properties of thermoplastic aligners and the absence of skeletal anchorage, their use is more appropriate for moderate transverse widening rather than extensive skeletal correction.^40^ Digital overcorrection, particularly in the molar region, may enhance expansion accuracy although further studies are needed to confirm its long-term periodontal implications.^32^,^49^

For patients presenting with larger transverse discrepancies or increased cortical bone density, hybrid mechanics such as combining aligners with limited fixed attachments or skeletal anchorage may enhance posterior control and torque management.^20^,^50^ Continuous monitoring of arch form and inclination during therapy remains essential to minimize excessive tipping and maintain functional occlusion. When applied within physiologic limits, aligner-based expansion represents a minimally invasive option for dentoalveolar correction while preserving esthetics and oral hygiene.

Future investigations should incorporate three-dimensional imaging modalities, such as cone-beam computed tomography, to differentiate dental from skeletal contributions during aligner-mediated expansion. Larger, multicentric cohorts and extended follow-up periods are necessary to evaluate long-term stability. Integrating finite-element modelling and artificial-intelligence-based predictive algorithms may further refine digital planning by estimating optimal overcorrection ratios and identifying patient-specific biomechanical variables influencing transverse movement. Such advances could enhance the accuracy of virtual setups and reduce the need for mid-course refinements.

## Limitations

This study has several limitations. The sample size was modest, and the 16-week observation period may not fully capture long-term treatment behavior or post-treatment stability. The absence of cone-beam computed tomography limited differentiation between skeletal and dentoalveolar components of expansion, restricting interpretation primarily to dental changes. Because only skeletally mature adults were included, extrapolation to growing or mixed-dentition populations should be made with caution. Additionally, exclusion of participants with documented non-compliance may have introduced selection bias, as the reported expansion accuracy reflects outcomes under controlled adherence conditions and may not fully represent real-world variability in aligner wear. Although digital models provide high-dimensional accuracy, they do not permit direct assessment of alveolar bone remodeling or cortical plate adaptation. Future multicenter investigations incorporating larger cohorts and volumetric imaging modalities are warranted to enhance generalizability and further elucidate the relative dental and skeletal components contributing to aligner-mediated transverse changes.

## Conclusions

Clear aligner therapy produced measurable, and symmetrical dentoalveolar maxillary expansion in skeletally mature adults; however, the magnitude of transverse change was consistently lower than digitally planned values. Expansion appeared to occur predominantly through controlled buccal tipping rather than bodily translation. These findings indicate that clear aligners are suitable for achieving mild to moderate dentoalveolar transverse widening; however, calibrated digital overcorrection, particularly in posterior regions, may be considered to improve alignment between virtual planning and clinical expression.

## Data Availability

The datasets generated and/or analyzed during the current study are available from the corresponding author upon reasonable request. All digital models and measurement records are archived in the institutional repository of Santosh Deemed to be University (Ref: SU-ORTHO-DATA-CAT-2024) and can be made available for verification in accordance with institutional data protection policies.

## References

[1] Dakhil NB, Salamah FB. The diagnosis methods and management modalities of maxillary transverse discrepancy. *Cureus*. 2021;13(12):e20482. 35047300 10.7759/cureus.20482 PMC8760022

[2] da Silva Filho OG, Santamaria M, Filho LC. Epidemiology of posterior crossbite in the primary dentition. *J Clin Pediatr Dent*. 2007;32(1):73–15. doi:10.17796/jcpd.32.1.h53g027713432102 18274476

[3] Andrucioli MCD, Matsumoto MAN. Transverse maxillary deficiency: treatment alternatives in face of early skeletal maturation. *Dental Press J Orthod*. 2020;25:70–79. doi:10.1590/2177-6709.25.1.070-079.bbo 32215481 PMC7077945

[4] Ottaviano G, Maculan P, Borghetto G, et al. Nasal function before and after rapid maxillary expansion in children. *Int J Pediatr Otorhinolaryngol*. 2018;115:133–138. doi:10.1016/j.ijporl.2018.09.029 30368373

[5] McNamara JA, Lione R, Franchi L, et al. The role of rapid maxillary expansion in oral and general health. *Prog Orthod*. 2015;16(1):33. doi:10.1186/s40510-015-0105-x 26446931 PMC4596248

[6] Kim S-H, Kim KB. Advances in maxillary transverse deficiency treatment. *Semin Orthod*. 2025;31(2):177–178. doi:10.1053/j.sodo.2024.12.004

[7] Pinheiro FHSL, Garib DG, Janson G, Bombonatti R, Freitas MR. Longitudinal stability of rapid and slow maxillary expansion. *Dental Press J Orthod*. 2014;19(6):70–77. doi:10.1590/2176-9451.19.6.070-077.oar 25628082 PMC4347413

[8] Kulshrestha R, Parkar A, Vibhute P, et al. Methods of gaining space: a review. *ES J Dent Sci*. 2020;1(2):1012.

[9] Choi J-Y, Kang Y-G, Kim S-J, Kim K-A. Functional outcomes of maxillary expansion. *Semin Orthod*. 2025;31(2):207–213. doi:10.1053/j.sodo.2024.08.003

[10] Sicca N, Benedetti G, Nieri A, et al. Side effects of MARPE vs SARPE in adult patients: a scoping review. *Dent J*. 2025;13(2):47. doi:10.3390/dj13020047 PMC11854554 39996921

[11] da Silva J, Moraes R, Vedovello SA, de Oliveira TC. Side effects of MARPE vs SARPE. *Ann Orthod Periodontics Spec*. 2025;5(1):1–20. doi:10.51847/jiwL0hv4Wq

[12] Weissheimer A, de Menezes LM, Mezomo M, et al. Immediate effects of RME with haas and hyrax expanders. *Am J Orthod Dentofacial Orthop*. 2011;140(3):366–376. doi:10.1016/j.ajodo.2010.07.025 21889081

[13] Badabaan G. *Clear aligner treatment by general dental practitioners in UK: a mixed-methods study* [Thesis]. Queen Mary University of London; 2025.

[14] Bichu YM, Alwafi A, Liu X, et al. Advances in clear aligner materials. *Bioact Mater*. 2023;22:384–403. doi:10.1016/j.bioactmat.2022.10.006 36311049 PMC9588987

[15] Macrì M, D’Albis V, Marciani R, et al. Sustainable orthodontics & aligners. *Materials*. 2024;17(17):4171. doi:10.3390/ma17174171 39274561 PMC11395928

[16] Muro MP, Caracciolo ACA, Patel MP, et al. Effectiveness of clear aligners. *Int Orthod*. 2023;21(2):100755. doi:10.1016/j.ortho.2023.100755 37086643

[17] D’Antò V, Valletta R, Di Mauro L, et al. Predictability of transverse changes with aligners. *Materials*. 2023;16(5):1910. doi:10.3390/ma16051910 36903025 PMC10004392

[18] AlMogbel A. Clear aligner therapy review. *J Orthod Sci*. 2023;12(1):37. doi:10.4103/jos.jos_30_23 37881665 PMC10597356

[19] Galluccio G, De Stefano AA, Horodynski M, et al. Expansion accuracy with aligners. *Int J Environ Res Public Health*. 2023;20(5):4634. doi:10.3390/ijerph20054634 36901642 PMC10002100

[20] Li N, Wang C, Yang M, et al. FEM of expansion with aligners. *Front Bioeng Biotechnol*. 2024;12:1424319. doi:10.3389/fbioe.2024.1424319 38983604 PMC11231920

[21] Naoum S, Lee R. Predictability of clear aligner movement. *Semin Orthod*. 2025.

[22] Kalekar AA, Manchanda J, Chavan S, et al. Maxillary expansion with aligners: meta-analysis. *Aust Orthod J*. 2024;40:111–129.

[23] Vas NV, Ramasamy N, Harikrishnan S, et al. FEM analysis of TAD-assisted advancement. *Turk J Orthod*. 2025;38(2):97. doi:10.4274/TurkJOrthod.2025.2024.105 40619949 PMC12236120

[24] de-la-Rosa-Gay C, Valmaseda-Castellón E, Figueiredo R, et al. Predictability factors in expansion. *Clin Oral Investig*. 2025;29(5):257. doi:10.1007/s00784-025-06328-y PMC12011961 40257582

[25] Castroflorio T, Sedran A, Parrini S, et al. Predictability of aligner movements. *Prog Orthod*. 2023;24(1):2. doi:10.1186/s40510-022-00453-0 36642743 PMC9840984

[26] Zhou N, Guo J. Efficiency of upper arch expansion with Invisalign. *Angle Orthod*. 2020;90(1):23–30. doi:10.2319/022719-151.1 31368778 PMC8087062

[27] Tien R, Patel V, Chen T, et al. Predictability of expansion with Invisalign. *Am J Orthod Dentofacial Orthop*. 2023;163(1):47–53. doi:10.1016/j.ajodo.2021.07.032 36195544

[28] Mo C, Jones O, Firth F. Digital workflow in orthodontics. *N Z Dent J*. 2025;121(2):73–80.

[29] Lee KC. Advancing orthodontic treatment through digital strategies and AI. *Semin Orthod*. 2025;31.

[30] Eggmann F, Blatz MB. Recent advances in intraoral scanners. *J Dent Res*. 2024;103(13):1349–1357. doi:10.1177/00220345241271937 39382136 PMC11633065

[31] Haude A-M, Lehmann T, Hennig C-L, Jacobs C. 2D vs 3D imaging in orthodontics. *J Orofac Orthop*. 2025;1–18. 10.1007/s00056-024-00574-7 39976657

[32] Keilig L, Fittgen A, Schneider H, et al. Accuracy of aligner digital planning. *J Clin Med*. 2024;13(8):2298. doi:10.3390/jcm13082298 38673571 PMC11051260

[33] Regragui S, Dakka A, Regragui F, Bouklouz A, Zaoui F. Which method to measure dentomaxillary discrepancy? *Int Orthod*. 2010;8(1):64–77. doi:10.1016/j.ortho.2009.12.005 20350717

[34] Sawchuk D, Currie K, Vich ML, Palomo JM, Flores-Mir C. Diagnostic methods for transverse deficiencies: a systematic review. *Korean J Orthod*. 2016;46(5):331–342. doi:10.4041/kjod.2016.46.5.331 27668196 PMC5033772

[35] Zhao R, Li M, Hu L, Fan J, Lai W. Changing clear aligners every 10 or 14 days? A randomized controlled trial. *Aust Orthod J*. 2023;39(1):1–12.

[36] Moreira DD, Gribel BF, Torres GDR, et al. Reliability of measurements on virtual models. *Braz J Oral Sci*. 2014;13(4):297–302. doi:10.1590/1677-3225v13n4a11

[37] Koo TK, Li MY. ICC guidelines. *J Chiropr Med*. 2016;15(2):155–163. doi:10.1016/j.jcm.2016.02.012 27330520 PMC4913118

[38] Staley RN, Reske NT. Management of Posterior Crossbites. In: *Essentials of Orthodontics Diagnosis and Treatment*. Wiley Online Library; 2011:113–133.

[39] Rossini G, Parrini S, Castroflorio T, et al. Efficacy of clear aligners. *Angle Orthod*. 2015;85(5):881–889. doi:10.2319/061614-436.1 25412265 PMC8610387

[40] Morales-Burruezo I, Gandía-Franco JL, Cobo J, et al. Invisalign expansion predictability. *PLoS One*. 2020;15(12):e0242979. doi:10.1371/journal.pone.0242979 33301484 PMC7728268

[41] Loberto S, Pavoni C, Fanelli S, et al. Expansion with aligners in mixed dentition. *BMC Oral Health*. 2024;24(1):694. doi:10.1186/s12903-024-04435-y 38879479 PMC11179342

[42] Yao S, Jiang W, Wang C, et al. Torque control during aligner expansion: FEM. *Front Bioeng Biotechnol*. 2023;11:1120535. doi:10.3389/fbioe.2023.1120535 37324442 PMC10267454

[43] Suzuki S, Nakano H, Kataoka Y, Maki K. Aligner force during lateral expansion. *Clin Invest Orthod*. 2023;82(2):67–74. doi:10.1080/27705781.2023.2179814

[44] Zhang Y, Hui S, Gui L, Jin F. FEM of upper arch expansion with aligners. *BMC Oral Health*. 2023;23(1):891. doi:10.1186/s12903-023-03655-y 37986060 PMC10662809

[45] Albertini P, Mazzanti V, Mollica F, et al. Stress relaxation properties of orthodontic aligner materials. *Bioengineering*. 2022;9(8):349. doi:10.3390/bioengineering9080349 36004874 PMC9405504

[46] Weir T. Clear aligners in orthodontics. *Aust Dent J*. 2017;62(S1):58–62. doi:10.1111/adj.12480 28297094

[47] Urooge A, Patil BA. Maxillary sinus sexual dimorphism. *J Clin Diagn Res*. 2017;11(3):ZC67. doi:10.7860/JCDR/2017/25159.9584 PMC5427439 28511513

[48] Oikonomou E, Foros P, Tagkli A, et al. Oral health in aligner vs fixed appliances. *Oral Health Prev Dent*. 2021;19:b2403661. doi:10.3290/j.ohpd.b2403661 34874143 PMC11641221

[49] de Figueiredo MA, Romano FL, Feres MFN, et al. Alveolar bone changes after aligner expansion. *Korean J Orthod*. 2023;53(4):264–275. doi:10.4041/kjod22.243 37497583 PMC10387425

[50] Martins RP. Achieving optimal orthodontic results with hybrid strategies. *Dental Press J Orthod*. 2025;30(4):e25spe4. doi:10.1590/2177-6709.30.4.e25spe4

